# Elevated expression of MMP-13 and TIMP-1 in head and neck squamous cell carcinomas may reflect increased tumor invasiveness

**DOI:** 10.1186/1471-2407-4-42

**Published:** 2004-08-03

**Authors:** Nil Culhaci, Kubilay Metin, Eray Copcu, Emel Dikicioglu

**Affiliations:** 1 Department of Pathology, Adnan Menderes University, Faculty of Medicine, Aydin, Turkey; 2 Department of Ear, Nose and Throat, Adnan Menderes University, Faculty of Medicine, Aydin, Turkey; 3 Department of Plastic and Reconstructive Surgery, Adnan Menderes University, Faculty of Medicine, Aydin, Turkey

## Abstract

**Background:**

Matrix metalloproteinases [MMPs], which degrade the extracellular matrix, play an important role in the invasion and metastasis of squamous cell carcinomas. One MMP, MMP-13, is thought to play a central role in MMP activation. The purpose of this study was to investigate MMP-13 and TIMP-1 expression in squamous cell carcinomas of the head and neck and to relate these levels of expression to histologic patterns of invasion.

**Methods:**

This study included T1 lesions obtained via biopsy from the larynx, tongue, and skin/mucosa of 78 patients with head and neck squamous cell carcinomas. The relationship between expression of MMP-13 and TIMP-1 and the mode of tumor invasion [MI] was evaluated immunohistochemically, using breast carcinoma tissue as a positive control.

**Results:**

Increased expression was observed in highly invasive tumors, as reflected by the significant correlation between the degree of staining for MMP-13 or TIMP-1 and MI grade [p < 0.05]. There was no significant relationship between the degree of staining for MMP-13 or TIMP-1 and patient age, sex, tumor site, or tumor histologic grade. In addition, levels of staining for MMP-13 did not correlate with levels of staining for TIMP-1.

**Conclusion:**

The expression of MMP-13 and TIMP-1 appears to play an important role in determining the invasive capacity of squamous cell carcinomas of the head and neck. Whereas additional studies are needed to confirm these findings, evaluating expression of these MMPs in small biopsy samples may be useful in determining the invasive capacity of these tumors at an earlier stage.

## Background

The invasion of surrounding tissues by neoplastic cells is one of the most important steps in tumor progression. Proteolytic enzymes such as matrix metalloproteinases [MMPs] contribute to tumor expansion by degrading components of the extracellular matrix [ECM]. MMPs are a 21-member family of zinc-dependent endopeptidases, which are capable of degrading most ECM components including collagen, elastin, fibronectin, and gelatin. Whereas this facilitates processes such as wound healing, enhanced MMP activity has been observed in a variety of pathologic conditions including osteoarthritis, rheumatoid arthritis, cardiovascular disease, and neoplasia [1-6].

MMPs can be divided into subgroups, which include collagenases, stromelysins, stromelysins-like MMPs, gelatinases, membrane-type MMPs, and others [5]. MMP-13 [collagenase 3] is a member of the collagenase family. It was first identified in human breast cancer and is active against a wide variety of ECM components [7]. MMP-13 also plays a central role in the MMP activation cascade, both activating and being activated by several MMPs [8]. Elevated MMP-13 expression has been found in a number of different malignancies, and expression has been related to tumor behavior and patient prognosis [7,9,10].

MMPs can be inactivated by specific tissue inhibitors of matrix metalloproteinases [TIMPs]. Thus far, four different TIMPs [TIMP-1, -2, -3, -4 ] have been identified [11] and implicated in connective tissue turn-over and remodeling. TIMPs inhibit MMPs by binding to them and forming non-covalent complexes [5]. Recent studies have linked increased MMP expression and decreased TIMP expression with tumor aggressiveness; however, other studies have shown overexpression of TIMPs in some patients with advanced tumors [12,13].

Squamous cell carcinoma [SCC] of the head and neck region is a major problem. Many studies have shown that MMPs are expressed in SCCs [5,14,15], with particular involvement by MMP-2 and MMP -9 [5,13,16]. MMP-13, which plays a central role in MMP activation, has also been shown to be highly expressed in head and neck SCCs [HNSCCs] [5]. As MMPs appear to be essential for tumor invasion and metastatic spread, characterizing their expression might help to determine a patient's treatment or prognosis. Therefore, we investigated the expression of MMP-13 and TIMP1 in biopsy specimens of HNSCCs to determine the relationship between the expression of these proteins and the mode of tumor invasion.

## Methods

### Patient and tumor characteristics

Between 1998 and 2003, we obtained 78 incisional and excisional biopsy samples from the primary tumors of 78 patients with invasive SCC who were admitted to the Head and Neck Surgery and Plastic Surgery Departments. Fifty-four men and 24 women were included in the study, and the mean patient age was 64 years [range: 26 to 88 years]. T1 stage tumors from the larynx, tongue, and skin or mucosa of the face, cheek, lip, nose, and ear were studied. Table 1 summarizes clinical characteristics of the patients. None had undergone prior chemotherapy or radiotherapy. All patients provided informed consent to participate, and the study protocol was approved by our institution's ethics committee.

Tumors were histologically graded as well [G1], moderately [G2], or poorly [G3] differentiated [Table 1]. The mode of tumor invasion [MI] was graded as follows: grade 1, well-defined border; grade 2, less-marked border; grade 3, groups of cells with no distinct border; grade 4, diffuse invasion [a, cordlike; b, widespread] [Table 1].

### Immunohistochemical procedures

The most representative block of tumor tissue was chosen in each case, and 5-μm sections were obtained and mounted on poly-L-lysine-coated slides for immunohistochemical staining. A standard streptavidin-biotin immunoperoxidase method was used for immunostaining with MMP-13 [7 ml, MS-825-R7, Ready-to-use, Neomarkers, USA] and TIMP-1 [7 ml, MS-608-R7, Ready-to-use, Neomarkers, USA] antibodies. The tissue sections were deparaffinized in xylene, rehydrated in an alcohol series, and immersed in distilled water. The sections were then boiled in a citrate buffer solution [10 mmol/L, pH= 6.0] in a microwave oven X3 for 10 minutes for antigen retrieval of both the MMP-13 and TIMP-1 antibodies. Endogenous peroxidase activity was blocked by exposing sections to a 0.3% solution of hydrogen peroxidase in phosphate-buffered saline [PBS] for 10 minutes at room temperature. After the sections had been rinsed with TRIS buffer, primary antibodies were applied for 60 minutes at room temperature followed by TRIS buffer. Linking antibody and streptavidin peroxidase complex were then added consecutively for 10 minutes at room temperature, and sections were washed again in TRIS buffer. After applying AEC chromogen, the sections were washed in deionized water, counterstained and mounted. Breast carcinoma tissue (which showed positive staining) was used as positive control during the evaluation of MMP-13 and TIMP-1 immunostaining.

### Evaluation/scoring of the staining

**Figure 1:**
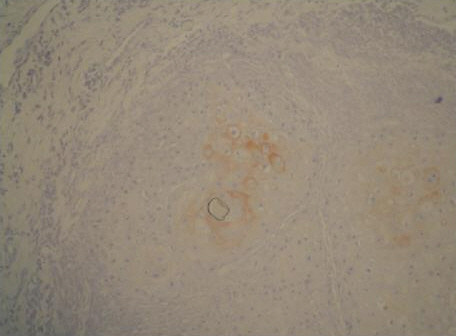
Moderate MMP-13 staining of the tumor cells in MI grade 2 SCC (anti-MMP-13, original magnification, × 100)

**Figure 2:**
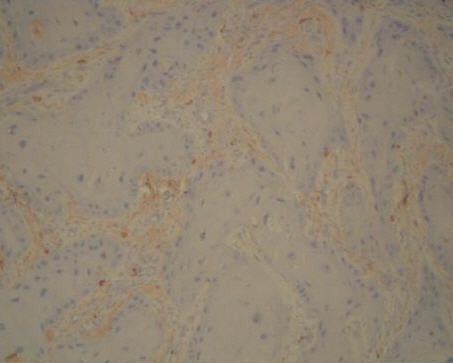
Moderate TIMP-1 staining of the stromal cells in MI grade 3 SCC (anti-TIMP-1, original magnification, × 200)

**Figure 3:**
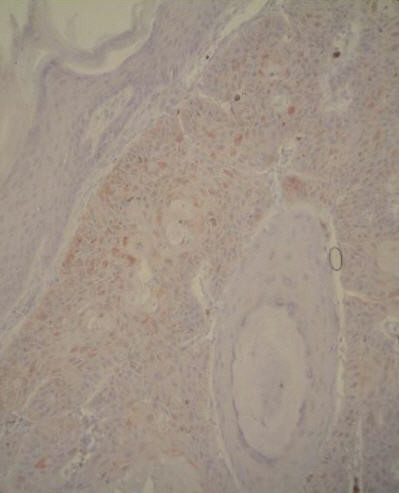
Normal epithelial cells at the top were not stained with MMP-13 (anti-MMP-13, original magnification, × 200)

One pathologist [NC] evaluated the stained slides associated with each case. The degree of staining for MMP-13 and TIMP-1 was scored as follows: 0, no staining of the tumor or stromal cells; 1+, weak [< 50%] positive staining of the tumor cells and/or weak staining of stromal cells; 2+, moderate [> 50%] positive staining of the tumor cells and/or moderate staining of stromal cells; 3+, extensive staining of the tumor cells and/or strong staining of stromal cells [Figs. 1,2] [12]. No normal epithelial cells were stained [Fig. 3].

### Follow-up

If necessary, surgery or radiotherapy was performed after the diagnosis. All patients were followed-up postoperatively for a mean interval of 20 months [range: 7 to 36 months]. During this period, 2 of 78 [2.6%] patients died from their tumors [one in larynx, one in tongue], and 1 patient died of an unrelated cause. Seventy-five of 78 [96.2%] patients were free of disease at the end of the follow-up period.

### Statistical analysis

The chi-square test was used for statistical analysis. Data were analyzed using SPSS for Windows 10,0. A p level <0.05 was considered to be statistically significant.

## Results

Although expression of cytoplasmic MMP-13 was primarily detected in tumor cells, it also was seen in stromal cells. MMP-13 expression was evident in tissue from 44 [56.4%] of the 78 patients. High levels of MMP-13 were noted in highly invasive tumors. There was no difference in the amount of staining between tumor centers or margins. Significant TIMP-1 expression was detected in tissue from 42 [53.8%] patients. Prominent labeling was confined to the stroma surrounding the tumor cells.

Table 2 shows the relationship between MMP-13 expression and MI in the 78 biopsy specimens. Increased expression of MMP-13 was observed in highly invasive tumors [MI grades 4a and b]. The relationship between MMP-13 expression and MI grade was statistically significant [p < 0.05].

Table 3 summarizes the relationship between TIMP-1 expression and MI in the 78 biopsy specimens. Increased expression of TIMP-1 was observed in highly invasive tumors. The relationship between TIMP-1 expression and MI grade was statistically significant [p < 0.05].

There was no statistically significant relationship between the degree of staining for MMP-13 or TIMP-1 and patient age, sex, tumor site, or histologic grade. Also, the degree of staining for MMP-13 did not significantly correlate with the degree of staining for TIMP-1.

## Discussion

As tumor invasion and metastasis affect a patient's prognosis, it is important to predict the invasive potential of tumors such as SCCs at an early stage. Several steps are involved in the invasion and metastasis of malignant cells including the attachment of cells to the ECM, the breakdown of matrix components, cell detachment, and migration of cells through the degraded matrix. This complex process requires several proteases, and the local balance between these proteases and protease inhibitors appears to be crucial. The MMPs represent one family of degrading proteases, which are expressed in various tumors. Many studies have shown that MMPs, especially MMP-2, -3, -9, are expressed in SCCs [5,12,16-18]. In the present study, we characterized the expression of one MMP–MMP-13, which plays a key role in the MMP activation cascade–and one inhibitor of MMPs, TIMP-1, in HNSCCs. Our finding that expression of both proteins was upregulated in markedly invasive tumors is consistent with prior reports and may have important therapeutic and prognostic implications.

The expression of several MMPs has been investigated in SCCs including MMP-9 and MMP-13. MMP-9 expression was found to correlate the most strongly with advanced pathological stages, and patients with HNSCCs also may have elevated serum concentration of MMP-9 [17,19]. Furthermore, expression of some MMPs such as MMP-9 appears to vary not only between the primary tumor and sites of lymph node metastasis, but also between the early and late stages of lymph node metastasis [16].

MMP-13 is a member of the collagenase family, which degrades fibrillar collagens of types I, II, III, IV, X, and XIV, tenascin, fibronectin, aggrecan, versican, and fibrillin-I. It is now accepted that MMP-13 plays a key role in the MMP activation cascade, both activating and being activated by several MMPs [MT1-MMP, MMP-2, MMP-3] [8,11]. MMP-13 expression is also susceptible to stimulation by several cytokines and growth factors such as TNF-α and TGF-β [11,15]. Elevated MMP-13 expression has been documented in numerous malignancies [e.g., breast carcinoma, colorectal cancer, vulvar SCC, skin SCC, HNSC, BCC (in focal areas of keratinized cells)] and associated with tumor behavior and patient prognosis [1,7-10,20]. Both small and large tumors appear to express MMP-13, with expression being the most prevalent in undifferentiated tumors [11].

MMP-13 expression has also been observed in transformed, but not primary, human epidermal keratinocytes [11,15]. Although MMP-1, -2, -3 have been detected in actinic keratosis [12,21], premalignant and benign tumors were mostly negative for MMP-13 in one study [20]. It is thought that MMP-13, alone, can markedly enhance the invasive capacity of malignant cells [22]. In transformed keratinocytes, p 53 plays an important role in suppressing MMP-13 expression [23]. Whereas some investigators have not found a significant relationship between MMP-13 expression and HNSCC behavior [17], we found significant MMP-13 expression in highly invasive tumors; this finding suggests that MMP-13 likely plays a role in regulating tumor invasion. In regard to location, cellular events at the tumor-stromal interface are thought to be more closely related to metastatic potential than events at the tumor's center [17,24]. Others have reported that MMP-13 is not only expressed by tumor cells in the invading periphery of most SCCs but also by stromal cells in a subset of tumors [15]. In the present study, we found no difference in levels of MMP-13 expression between tumor centers or margins.

TIMP-1 is another protein that has been implicated in tumor growth. TIMP-1 mRNA has been detected in well-differentiated cancer cells, proliferated keratinocytes, and endothelial cells [2,14], and TIMP-1 overexpression has been observed in almost every case of HNSCC [24]. Whereas some investigators have reported that increased expression of TIMP-1 and TIMP-2 correlates with less aggressive tumors, others have reported the opposite finding [12,13]. In the present study, TIMP-1 expression was significantly increased in markedly invasive tumors, suggesting that TIMP-1 also plays a role in regulating tumor invasiveness.

In some studies, no association between MMP-13 and TIMP-1 expression and any clinicopathological variables was found, or TIMP expression did not correlate with tumor growth [13,17]. Our results are not compatible with these studies. However, we did fail to identify any statistically significant relationship between the degree of staining for MMP-13 or TIMP-1 and the patient's age, sex, tumor site, or tumor histologic grade. Also, there was no statistically significant correlation between the degree of staining for MMP-13 and TIMP-1. This suggests that the balance between MMP and TIMP expression may not be as important to tumor invasion as their overexpression; that is, MMP and TIMP may play important separate roles in tumor invasion in which they act via different mechanisms.

Because of the relatively short follow-up interval, we are unable to demonstrate whether MMP-13 and TIMP-1 expression is related to long-term survival. However, 75 of our 78 patients were free of disease after a mean follow-up interval of 20 months. As poor outcomes are the result of local recurrences and distant metastasis, factors that reflect the invasive and metastatic potential of SCCs, such as MMPs, could be helpful in predicting patient prognosis. Thus, further investigation of MMPs may not only clarify their role in tumor invasion but also may facilitate the development of new therapeutic approaches.

## Conclusion

The results of this study suggest that MMP-13, which plays a central role in MMP activation, and the MMP inhibitor, TIMP-1, help regulate the invasiveness of HNSCCs. Although other methods [e.g., Western blots] are needed to confirm these findings, examination of MMP-13 and TIMP-1 expression in small biopsy samples might be useful in determining the invasive capacity of these tumors at an earlier stage.

## Competing interests

None declared.

## Authors' contributions

NC drafted and wrote the manuscript.

KM and EC performed the surgery and follow-up of the patients.

ED participated in the design and coordination of the study.

All authors read and approved the final manuscript.

## Pre-publication history

The pre-publication history for this paper can be accessed here:



## Figures and Tables

**Table 1:** Clinicopathologic features

***Characteristic***	***Number of cases ***
Age	
< 60 years	27
> 60 years	51
Sex	
Male	54
Female	24
Tumor site	
Larynx	26
Tongue	8
Skin and mucosa	44
Histologic grade	
Differentiated (G1)	39
Poorly differentiated (G2, G3)	39
Mode of tumor invasion	
Well defined (1 + 2)	18
No distinct border (3)	43
Diffuse invasion (4a + 4b)	17

**Table 2:** Relationship between level of MMP-13 expression and MI

	***MMP-13 staining***		
	
***Grade of tumor invasion***	Negative/weak (0 + 1)	Moderate/ extensive (2 + 3)	**Total**
**1 (1 + 2)**	15 (83.3%)	3 (16.7%)	**18**
**2 (3)**	14 (32.6%)	29 (67.4%)	**43**
**3 (4a + 4b)**	5 (29.4%)	12 (70.6%)	**17**
Total	**34**	**44**	**78**

MI: mode of tumor invasion (expressed as grade). p values: 0.001

**Table 3:** Relationship between level of TIMP-1 expression and MI

	***TIMP-1 staining***		
	
***Grade of tumor invasion***	Negative/weak (0 + 1)	Moderate/ extensive (2 + 3)	**Total**
**1 (1 + 2)**	13 (72.2%)	5 (27.8%)	**18**
**2 (3)**	16 (37.2%)	27 (62.8%)	**43**
**3 (4a + 4b)**	7 (41.2%)	10 (58.8%)	**17**
Total	**36**	**42**	**78**

MI: mode of tumor invasion (expressed as grade). p values: 0.03
